# Setting global research priorities for integrated community case management (iCCM): Results from a CHNRI (Child Health and Nutrition Research Initiative) exercise

**DOI:** 10.7189/jogh.04.020413

**Published:** 2014-12

**Authors:** Kerri Wazny, Salim Sadruddin, Alvin Zipursky, Davidson H. Hamer, Troy Jacobs, Karin Kallander, Franco Pagnoni, Stefan Peterson, Shamim Qazi, Serge Raharison, Kerry Ross, Mark Young, David R. Marsh

**Affiliations:** 1 Centre for Global Child Health, the Hospital for Sick Children, Toronto, Canada; 2 Save the Children, Fairfield, CT, USA; 3 Programme for Global Paediatric Research, the Hospital for Sick Children, Toronto, Canada; 4 Zambia Center for Applied Health Research and Development, Lusaka, Zambia; 5 Center for Global Health and Development, Boston University, Boston, MA, USA; 6 Department of International Health, Boston University School of Public Health, Boston, MA, USA; 7 Global Health Bureau, United States Agency for International Development, Washington DC, USA; 8 Malaria Consortium, London, UK; 9 Department of Public Health Sciences, Karolinska Institutet, Stockholm, Sweden; 10 Makerere University School of Public Health, Kampala, Uganda; 11 Global Malaria Programme, World Health Organization, Geneva, Switzerland; 12 International Women’s and Children’s Health, Uppsala University, Sweden; 13 Global Health, Karolinska Institutet, Sweden; 14 Department of Maternal, Newborn, Child and Adolescent Health, World Health Organization, Geneva, Switzerland; 15 John Snow, Inc., Washington DC, USA; 16 Maternal and Child Health Integrated Project (MCHIP), Washington, DC, USA; 17 Programme Division, United Nations Children’s Fund, New York, USA

## Abstract

**Aims:**

To systematically identify global research gaps and resource priorities for integrated community case management (iCCM).

**Methods:**

An iCCM Child Health and Nutrition Research Initiative (CHNRI) Advisory Group, in collaboration with the Community Case Management Operational Research Group (CCM ORG) identified experts to participate in a CHNRI research priority setting exercise. These experts generated and systematically ranked research questions for iCCM. Research questions were ranked using a “Research Priority Score” (RPS) and the “Average Expert Agreement” (AEA) was calculated for every question. Our groups of experts were comprised of both individuals working in Ministries of Health or Non Governmental Organizations (NGOs) in low– and middle–income countries (LMICs) and individuals working in high–income countries (HICs) in academia or NGO headquarters. A Spearman’s Rho was calculated to determine the correlation between the two groups’ research questions’ ranks.

**Results:**

The overall RPS ranged from 64.58 to 89.31, with a median score of 81.43. AEA scores ranged from 0.54 to 0.86. Research questions involving increasing the uptake of iCCM services, research questions concerning the motivation, retention, training and supervision of Community Health Workers (CHWs) and concerning adding additional responsibilities including counselling for infant and young child feeding (IYCF) and treatment of severe acute malnutrition (SAM) ranked highly. There was weak to moderate, statistically significant, correlation between scores by representatives of high–income countries and those working in–country or regionally (Spearman’s ρ = 0.35034, *P* < 0.01).

**Conclusions:**

Operational research to determine optimal training, supervision and modes of motivation and retention for the CHW is vital for improving iCCM, globally, as is research to motivate caregivers to take advantage of iCCM services. Experts working in–country or regionally in LMICs prioritized different research questions than those working in organization headquarters in HICs. Further exploration is needed to determine the nature of this divergence.

Approximately 6.6 million children die before their fifth birthday every year [1,2]. Together, pneumonia, diarrhoea and malaria accounted for approximately one third of these deaths [1,2], and many of these deaths are preventable. Although Millennium Development Goal 4 (MDG4) made reducing child deaths a global priority, calling for a two–thirds reduction of child deaths between 1990 and 2015, many countries are not on track to achieve this goal [2–6].

Diarrhoea and pneumonia, in particular, disproportionately affect impoverished and marginalized children who do not access treatment [1]. Existing interventions to prevent and treat childhood pneumonia, diarrhoea and malaria are efficacious [6]. Integrated community case management (iCCM) is a delivery strategy that utilizes community health workers (CHWs) to diagnose and treat multiple conditions, most commonly pneumonia, diarrhoea and malaria, in children under five. CHWs are based in the communities they serve, working as an easily accessible community–arm of a country’s existing health care system [7,8].

Major donors and non–governmental organisations (NGOs), including the World Health Organization (WHO) and the United Nations Children’s Fund (UNICEF) are promoting iCCM as a key strategy to reduce child mortality. CHWs have been lauded as “the world’s most promising health workforce resource for enabling health systems in resource–constrained settings to reduce the burden of disease from serious, readily preventable or treatable conditions” [5]. In addition to reducing pneumonia–specific mortality by 36% and malaria–specific mortality by 60%, a recent review found that CHWs can also effectively perform nutritional counselling activities [5,6].

Nepal’s iCCM program has contributed to one of the most rapidly declining child mortality rates in the world [5]. Conversely, despite having a national–level, well–funded, community health workers program, Pakistan has not achieved satisfactory reductions in child mortality [9,10]. This divergence of results highlights the need to understand programmatic factors to strengthen programs delivering iCCM [11,12]. In their call for identification of research priorities in iCCM, Hamer and colleagues emphasize the need for the integration of research and program implementation in addition to a focus on long–term outcomes [11].

While research priorities have previously been developed and published by the Global CCM Operations Research Group (CCM ORG), the development of these priorities was constrained because the advisors were global level iCCM experts and the research priorities were not systematically evaluated [11]. Thus, we applied the Child Health and Nutrition Research Initiative’s (CHNRI) method to identify and systematically evaluate research priorities for iCCM. The CHNRI method has identified research gaps and resource priorities in a variety of contexts, including global childhood diarrhoea, birth asphyxia and childhood pneumonia [13–17].To our knowledge, this is the first use of CHNRI to identify research gaps and resource priorities for a delivery strategy rather than an illness. Over seventy–five experts, representing academics, international organizations and Ministries of Health within countries already implementing iCCM participated in at least one of the steps of this exercise.

## METHODS

The CHNRI method was designed to assist policy makers and funders in identifying research gaps and resource priorities in a variety of contexts for health research, as well as the strengths and weaknesses of the research gaps identified. In the last decade, the CHNRI method has been widely used to identify research gaps in childhood illnesses, including global childhood diarrhoea, birth asphyxia and childhood pneumonia [13–17]. The exercise is comprised of four stages: (i) the context of the problem and the evaluation criteria are defined; (ii) technical experts generate and rank research questions against the proposed criteria; (iii) weighting of the evaluation criteria is decided through consultation with stakeholders; and, (iv) research priority scores are calculated for each research priority and agreement between experts is analyzed [18].

An iCCM CHNRI Advisory Group was formed to assist in the execution of this exercise. Together with the co–principal investigators (KW, SS, AZ), the Advisory Group attended a two–day meeting in New York where the criteria used in scoring was finalised and the final list of research questions to be scored was selected. A list of the iCCM CHNRI Advisory Group members and a detailed description of the activities during the New York meeting are presented in **Box 1****.**

Box 1 **Details of New York Meeting activities**

The members of the iCCM CHNRI Advisory Group and the co–PIs of the CHNRI exercise met in New York on May 1 and 2, 2013. Members of the iCCM CHNRI Advisory Group are: Shamim Qazi, David Marsh, Franco Pagnoni, Mark Young, Kerry Ross, Karin Kallander, Serge Raharison and Troy Jacobs. The co–principal investigators are: Kerri Wazny, Salim Sadruddin and Alvin Zipursky.

The members combined and eliminated duplicate research priorities, both from the original ORG list [11, 12] and from those submitted through the CHNRI exercise, leaving 119 research priorities. These priorities were scored on a scale of 1–5 (1 being highest, 5 being lowest), by all of the meeting participants. The average score for each priority was calculated, and priorities with a score higher than average (≥2.6) were retained for dissemination for scoring by the larger group of CHNRI participants.

The iCCM CHNRI Advisory Group and co–principal investigators also discussed the standard CHNRI criteria and modified CHNRI criteria used in a previous exercise [17], and finalized the criteria to be used in the iCCM CHNRI exercise. The group decided to use 4, rather than 5, criteria and to weight the criteria equally in the final analysis.

Finally, the group agreed to re–invite all those invited to submit research questions, regardless of whether they did, unless the participant expressed that they were unable to participate in the exercise.

### 1. Context of the problem and evaluation criteria are defined

We modified the CHNRI criteria used in a previous CHNRI exercise [17], yielding criteria more applicable for evaluating research questions for a delivery system. We chose the following four criteria: (i) answerability; (ii) research feasibility; (iii) deliverability; and, (iv) importance/potential impact. **Table 1** displays the specific questions used to evaluate the research questions under each criterion.

**Table 1:** Criteria for iCCM CHNRI exercise

Criterion	Sub–questions
Answerability	1. Would you say that the research question is well–framed? 2. Can a single study or a very small number of studies be designed to answer the research question? 3. Do you think that a study needed to answer the proposed research question would obtain ethical approval without major concerns?
Research Feasibility*	1. Is it likely that, in the context of interest, there will be sufficient capacity to carry out this research? 2. Is it feasible to provide the training required for staff to carry out the research in the context of interest? 3. Is the cost and time required for this research reasonable within the context of interest?
Deliverability	1. Taking into the account the level of difficulty with delivery of the potential intervention or delivery strategy (for example, need for change of attitudes and beliefs, supervision, transport infrastructure), would you say that this intervention or delivery strategy will be ***deliverable*** within the context of interest? 2. Taking into account the resources available to implement the intervention, would you say that the intervention or delivery strategy would be ***affordable*** within the context of interest? 3. Would government capacity and partnership be essential to ensure the intervention or delivery strategy would be ***sustainable***?†
Importance/ Potential Impact	1. Will the results of this research fill an important knowledge gap? 2. Are the results from this research likely to shape future planning and implementation? 3. Will the results from this research be relevant to most countries in the context of interest?

iCCM – integrated community case management, CHNR – Child Health and Nutrition Research Initiative

*For this criterion, the “context of interest” refers to countries that do, or would benefit from, implementation of iCCM.

†We eliminated this question from the calculation of the scores for Criterion 3, as described in the text.

### 2. Technical experts generate and rank research questions

We asked for members of the CCM ORG to nominate experts for participation in the exercise. We also included experts who participated in a previous CHNRI exercise who are involved in iCCM implementation or research [17]. Finally, we invited experts who were referred from others we invited to participate. In total, we invited 127 experts in iCCM to generate research questions for our CHNRI exercise. Experts represented international organizations, ministries of health within low– and middle–income countries, academia and non–governmental organizations. All materials, including instructions and research questions, were translated into French by a professional translator to ensure francophone country participation. In total, 75 experts submitted 366 research questions.

We combined the submitted research questions with those previously generated by the CCM ORG and thematically organized and discussed the research questions during the iCCM CHNRI Advisory Group Meeting [11]. The Advisory Group members removed duplicates, combined similar questions, and then rated each question from 1 to 5. We calculated average scores and selected the 61 questions with above average scores for evaluation by the experts.

We invited all experts who were initially approached to submit research questions, aside from those who declined participation, plus any additional experts we identified after our call for research questions. In total, we invited 133 experts to score the 61 questions. To reduce the possibility that the order of research questions would affect scoring, we made eight versions of the scoring sheet, using a random number generator to shuffle the question order. The scoring sheets were otherwise identical.

Each criterion contained three sub–questions. We asked experts to score 1 for yes, 0 for no and 0.5 if undecided. If the experts did not feel sufficiently knowledgeable to answer a particular question, they were instructed to leave the cell blank. Seventy–five experts returned completed scoring sheets.

### 3. Weighting of criteria is decided

Prior to scoring, we chose to weigh all criteria equally in the analysis, as we felt they were of equal importance.

### 4. Research Priority Scores and Average Expert Agreement are calculated

All returned scoring sheets were checked for errors and then scores were entered into a master calculation sheet. The Research Priority Score (RPS) and Average Expert Agreement (AEA) were calculated for each research question. The RPS is a mean score given, across criteria and scorers, for a particular research question. The AEA is the proportion of scorers who chose the mode (the most common score) for each research question.

After distributing scoring sheets to the experts, some of the experts raised concerns regarding the third question of criterion 3 (**Table 1**). This question asks whether government partnership will be necessary to ensure sustainability of the results of the research. A “yes” response indicates a positive answer for all other questions except this one. If government partnership is necessary to sustain research results, then a “yes” is a negative answer. Given the potential confusion over non–parallel construction, we excluded this question. Thus, we took the average scores for each criterion and then averaged those scores, weighting each criterion equally. For criterion 3, we calculated the average of questions 1 and 2 only. Calculating the RPS in this way, rather than taking the mean scores across the 11 sub–questions, allowed for each criterion to be weighted equally in the analysis.

We used the AEA rather than a Fleiss kappa statistic to calculate agreement among experts, which is in line with previous CHNRI exercises. Due to the large number of scorers and few scoring options, it is not possible to rule out chance with the Fleiss Kappa statistic even in cases with complete agreement. Although the AEA does not give an indication of statistical significance, we thought that policy makers and donors would find it more useful than the kappa statistic, as it can give a general idea of the degree of agreement between experts. The average expert agreement (AEA) was calculated as follows:

where q is a question that experts are being asked to evaluate competing research investment options, ranging from 1 to11

### 5. Comparative analysis of scores given by in–country or regional participants and those working in high–income countries or organizational headquarters

We stratified the responses received into those received by participants working in–country or regionally (LMIC Group) and those working in organizational headquarters or high–income countries (HQ/HIC Group). A list of our participants, their organizations and their categorizations can be found in **Online Supplementary Document(Online Supplementary Document), Table S1**. We calculated the RPS for each research question separately in these groups and used a Spearman’s Rho correlation coefficient to calculate the correlation of research questions’ ranks between these groups. Spearman’s Rho is used to determine the degree of correlation between two ranked sets of results. A correlation coefficient of 1 indicates high, positive correlation between two sets of results; a correlation coefficient of –1 indicates high, negative correlation between two sets of results and a correlation coefficient of 0 indicates no correlation.

## RESULTS

We invited 133 experts to score research questions; 75 returned completed scoring sheets. Three experts declined participation at this stage. We received nearly equal responses from participants working in–country or regionally (n = 36) and from those working in organizational headquarters or high–income countries (n = 39).

The range of RPS across the 61 questions was 64.58 to 89.31 (median = 81.43) out of a possible 100. The AEA ranged from 0.54 to 0.86 out of a possible 1.00. The top 20 research questions overall and their corresponding RPS and AEA scores are displayed in **Table 2****. Online Supplementary Document(Online Supplementary Document), Table S2** contains all the ranked research questions, their scores in each criterion and their RPS and AEA scores.

**Table 2:** Overall rank and research priority scores for top 20 research questions

Overall Rank	Research question	Research Priority Score (RPS)	Average Expert Agreement (AEA)
1	Assess perceptions of beneficiaries and levels of community satisfaction in CHWs capacity to diagnose and treat sick child (with malaria, pneumonia, diarrhoea and severe malnutrition) at the community level.	89.31	0.84
2	Identify and evaluate strategies for retention and motivation of CHWs.	89.08	0.86
3	Identify and evaluate strategies for improving referral between communities and health facilities, including referral compliance.	88.94	0.84
4	Identify determinants of non–use of iCCM services by caretakers and develop strategies to increase the uptake of iCCM.	88.89	0.84
5	Identify and evaluate new diagnostic tools for improved classification of pneumonia (ie, different ARI timers, respiratory counting beads, etc.) at the community level that are most appropriate for various cadres.	88.83	0.85
6	Evaluate the effectiveness of 3–day vs 5–day amoxicillin treatment regimens in Africa.	88.61	0.84
7	Identify and evaluate innovative strategies to improve community engagement and mobilization for CCM.	87.49	0.83
8	Evaluate the feasibility, effectiveness and impact of adding community–based infant and young child feeding (cIYCF) counseling skills to the CHW workload.	87.26	0.82
9	Identify the primary barriers to CHW supervision and develop and evaluate strategies to motivate CHW supervisors to provide continuous support to CHWs.	87.18	0.82
10	What is the impact of pre–referral antibiotics on treatment outcomes of possible serious bacterial infections?	86.52	0.80
11	Assess perceptions, understanding and motivating factors for caregivers on the need for prompt treatment for the sick child.	86.41	0.82
12	What is the impact of iCCM on health facility worker workload, by disease?	86.37	0.81
13	Develop and evaluate strategies (for example, innovative packaging of drugs) to improve compliance and uptake of treatment.	86.00	0.81
14	Identify and evaluate strategies to improve supervision and quality of care using mHealth technology.	85.85	0.81
15	Identify and evaluate effective and feasible strategies for maintaining quality of case management by CHWs.	85.56	0.82
16	Identify and evaluate strategies for, and costs of, supervising the CHW supervisor.	85.35	0.79
17	Develop and evaluate strategies for using mHealth technology to improve drug supply and logistics for the CHWs.	85.28	0.79
18	Evaluate the impact of iCCM on equity in access and use of basic health services.	85.14	0.80
19	Identify and evaluate the effectiveness and cost of various incentive schemes and strategies for CHWs.	84.81	0.79
20	Identify and evaluate strategies to improve integration of iCCM logistics (diagnostics and drug supply) to the central procurement and supply system at the community level.	84.41	0.79

CHW – Community Health Workers, iCCM – integrated community case management

Several of the top 20 research priorities overall involved increasing uptake of iCCM services, through community motivation and satisfaction (#1, #7), identification of determinants of non–use (#4), motivating factors for care seeking behaviour (#11) and other strategies to improve compliance and uptake (#13).

Strategies to improve motivation, retention, training and supervision of CHWs were a priority (questions #2, 9, 13, 16 and 19). Identifying and evaluating strategies for retention and motivation of CHWs was 2nd overall, scoring highly in importance/potential impact (0.92) and had high agreement between scorers (AEA = 0.86). Two of the top 20 questions (#9 and 14) emphasized the need for supervision and support of CHWs. mHealth technology was proposed as a tool to strengthen drug supply and logistics systems (#17).

Research questions involving adding responsibilities to CHWs’ workload also appeared in the top 25. Questions #8 and 21 address the feasibility, effectiveness and impact of adding counselling for infant and young child feeding (IYCF) and treatment of severe acute malnutrition (SAM), respectively.

Two of the top 10 research questions dealt specifically with pneumonia; question 5 asks to identify and evaluate new diagnostics specific to different cadres of health workers (eg, respiratory counting beads and ARI timers) and question 6 asks to evaluate 3–day vs 5–day amoxicillin treatment in Africa.

### Importance/potential impact

**Table 3** displays the top 10 questions in the importance/potential impact criterion. Question #15 overall (identify and evaluate feasible and effective strategies for maintaining CHWs’ quality of case management) ranked first, and question #15 (identify and evaluate new diagnostics for different CHW cadres) ranked second. Questions relating to adding newborn care and SAM to the CHWs’ workload, which were ranked 21^st^ and 24^th^ overall, respectively, both received scores of 0.90, but these questions’ scores in the answerability and deliverability criteria brought down their overall scores.

**Table 3:** Top 10 research priorities by importance/potential impact criterion

Importance/ Potential impact rank	Research question	Criterion 1 score	Criterion 2 score	Criterion 3 score	Criterion 4 score	Overall RPS	Overall Rank
1	Identify and evaluate effective and feasible strategies for maintaining quality of case management by CHWs.	0.77	0.90	0.83	0.93	85.56	15
2	Identify and evaluate new diagnostic tools for improved classification of pneumonia (ie, different ARI timers, respiratory counting beads, etc.) at the community level that are most appropriate for various CHW cadres.	0.87	0.90	0.85	0.93	88.83	5
3	Identify and evaluate strategies for retention and motivation of CHWs.	0.85	0.94	0.89	0.92	89.08	2
4	Evaluate effectiveness of 3–day vs 5–day oral amoxicillin treatment regimens in Africa.	0.83	0.88	0.92	0.91	88.61	6
5	What are the feasibility, impact and costs of adding newborn care (including PNS, home visits, treatment of infection and Caring for the Newborn and Children in the Community) to the iCCM package?	0.78	0.86	0.77	0.90	82.74	24
6	Develop safe and effective treatment strategies in settings where referral is not possible.	0.62	0.79	0.73	0.90	75.88	52
7	Evaluate the effectiveness and feasibility of delivering treatment for Severe Acute Malnutrition (SAM) through iCCM.	0.82	0.84	0.81	0.90	84.41	21
8	Identify the primary barriers to CHW supervisions and develop and evaluate strategies to motivate CHW supervisors to provide continuous support to the CHWs.	0.81	0.93	0.86	0.90	87.18	9
9	Identify and evaluate the effectiveness and cost of various incentive schemes and strategies for CHWs.	0.81	0.88	0.81	0.89	84.81	10
10	Identify and evaluate determinants of quality of CCM services, including characteristics of health systems (and supporting environment) that are most important for delivering high quality iCCM programs at–scale with limited external support.	0.74	0.86	0.84	0.89	83.22	22

RPS – research priority score, iCCM – integrated community case management

### Research feasibility and deliverability

We eliminated research questions that had scores of <0.8 in at least one of the following criteria: answerability, research feasibility and deliverability. We were aiming to investigate whether eliminating any research priorities that would either be difficult to design research studies to answer or to sustain in the post–research stage would change the results. In this analysis, six research priorities were eliminated from the top twenty–five overall. Research questions that ranked 7, 15, 18 and 22 in the overall list were eliminated due to low scores in the answerability category. Questions seventeen and twenty–four overall were eliminated due to a low score in the deliverability criterion and in both the deliverability and answerability criteria, respectively.

### In–country or regional participants vs participants from organizational headquarters or high–income countries

HQ/HIC Group scores (median 80.9, range: 63.3–91.1) were slightly lower than their LMIC counterparts (median 83.4, range: 63.5–93.1). The correlation of the research questions’ ranks between the HQ/HIC group and the LMIC group was weak to moderately positive, though statistically significant (Spearman’s ρ = 0.35045, *P* < 0.01).

While there was a divergence between research questions prioritized by the LMIC group vs the HQ/HIC group, both groups of experts scored the first overall research question highly; otherwise, many of the research questions with the highest level of agreement between groups were ranked in the middle or bottom by both groups.

Research questions and their corresponding ranks by informant are displayed in **Online Supplementary Document(Online Supplementary Document), Table S3**. Within this table, research questions with the highest level of disagreement between both groups are shaded; blue indicates research priorities that were ranked highly by the HQ/HIC group but not by those in the LMIC group and research priorities shaded in orange indicate the reverse. **Table 4** and **Table 5** display the top 5 research questions ranked by those working in organizational headquarter/HICs and by experts working in–country or regionally, respectively.

**Table 4:** Top 5 research priorities by organization HQ/HIC participants

HQ Rank	Research Question	Criterion 1	Criterion 2	Criterion 3	Criterion 4	HQ RPS	Overall Rank
1	Identify and evaluate new diagnostic tools for improved classification of pneumonia (ie, different ARI timers, respiratory counting beads, etc.) at the community level that are most appropriate for various CHW cadres.	0.89	0.92	0.87	0.96	91.09	5
2	Evaluate the effectiveness of 3–day vs 5–day amoxicillin treatment regimens in Africa.	0.83	0.90	0.92	0.94	89.83	6
3	Assess perceptions of beneficiaries and levels of community satisfaction in CHWs’ capacity to diagnose and treat sick children (with malaria, pneumonia, diarrhea and severe malnutrition) at the community level.	0.86	0.96	0.94	0.71	86.90	1
4	Identify and evaluate strategies for improving referral between communities and health facilities, including referral compliance.	0.79	0.94	0.85	0.90	86.86	3
5	Evaluate the effectiveness and feasibility of CHW’s use of pulse oximetry to identify children with severe pneumonia.	0.90	0.89	0.79	0.89	86.57	40

HQ/HIC – organizational headquarters or high–income countries, RPS – research priority score, iCCM – integrated community case management, CHW – community health worker

**Table 5:** Top 5 research priorities by LMIC participants

Country Rank	Research question	Criterion 1	Criterion 2	Criterion 3	Criterion 4	Country RPS	Overall Rank
1	Identify the primary barriers to CHW supervision and develop and evaluate strategies to motivate CHW supervisors to provide continuous support to CHWs.	0.89	0.98	0.92	0.94	93.10	9
2	Identify determinants of non–use of iCCM services by caretakers and develop strategies to increase the uptake of iCCM.	0.90	0.95	0.96	0.90	92.61	4
3	Identify and evaluate determinants of quality of CCM services, including characteristics of health systems (and supporting environment) that are most important for delivering high quality iCCM programs at–scale with limited external support.	0.85	0.94	0.94	0.98	92.55	22
4	Identify and evaluate strategies for retention and motivation of CHWs.	0.92	0.96	0.85	0.96	92.27	2
5	Identify and evaluate innovative strategies to improve community engagement and mobilization for CCM.	0.84	0.99	0.96	0.90	92.19	7

LMIC – low– and middle–income countries, RPS – research priority score, iCCM – integrated community case management, CHW – community health worker

**Figure 1:**
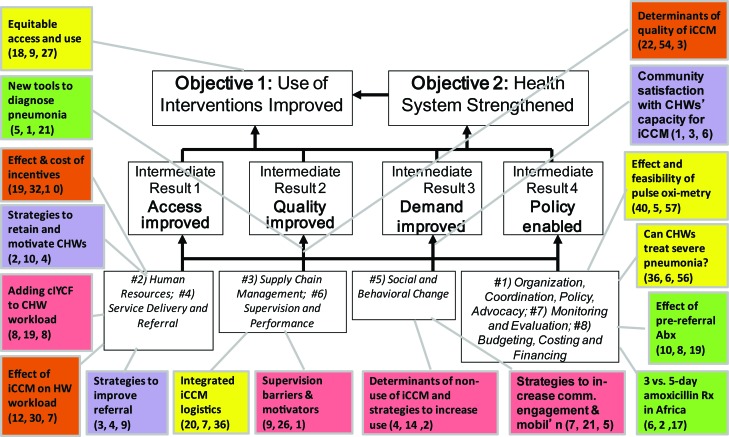
Evaluation Framework matched to CHNRI research priority “top 10” questions by list ranking: overall, HQ/HIC and LMIC. Number in parentheses: rank overall, HQ/HIC and LMIC, respectively. HQ/HIC – organizational headquarters or high–income countries; LMIC – low– and middle–income countries. Key: blue – top 10 in all questions, green – top 10 overall and in HQ/HIC, red – top 10 overall and in LMIC, orange – top 10 in LMIC only, yellow – top 10 in HQ/HIC only.

We mapped the research questions that appeared in the top 10 (either overall, in the LMIC list or the HQ/HIC list) to the iCCM evaluation framework [19]. Spread across the life cycle of the project, the framework (**Figure 1**) has 8 health system components: (i) organization, coordination, policy & advocacy; (ii) human resources; (iii) supply chain management; (iv) service delivery and referral; (v) social and behavioural change; (vi) supervision and performance; (vii) monitoring and evaluation; and, (viii) budgeting, costing and financing. The top 10 research questions in the LMIC and HQ/HIC lists appear in 7 of the 8 components. Listed under each research question in the Figure are the scores of that research question in the overall, HQ/HIC and LMIC lists, respectively. While some of the research questions have similar scores in all lists, the ranks of some of the research questions in the three lists are greatly divergent.

## DISCUSSION

This CHNRI exercise is the first one, to our knowledge, that uses the methodology to define research gaps and resource priorities for a delivery strategy, rather than a condition. Moreover, our exercise is the first to conduct a comparative analysis of the priorities of different groups of scorers. The results of this exercise will be important in defining the global research agenda for iCCM. Participation in this exercise was not limited to experts in HICs, but included experts based in low–income countries or at regional level, who are implementing or supporting iCCM programs.

Limitations to our exercise include representativeness of sampling and high non–response rate. Although we aimed to include as many experts as possible working in iCCM and issued several calls for names to be nominated, it is possible that we were not able to identify and invite all experts in iCCM. Furthermore, our response rate for the final scores was 56%. Conceivably, those who responded could be systematically different than those who did not. We believe the low response rate to be due to the time consuming nature of completing scoring sheets. However, our response rate was higher than those reported in previous CHNRI exercises [20–22].

Examining community perceptions and satisfactions with CHWs was the highest ranked research priority overall, and this research question had high scores in the deliverability and research feasibility criteria. However, it ranked 46^th^ in the importance/potential impact criteria with a score of 0.78, thus providing a good example of how the CHNRI method can be used to expose the strengths and weaknesses of a particular research question. Additionally, the research question with the highest score for importance/potential impact scored low in the answerability criterion, which indicates a potential for difficulty in designing a study to address it.

Experts working in LMICs prioritized research questions that were mainly operational or delivery–based, including strengthening CHW supervision, motivation and retention, increasing uptake of iCCM services by caretakers, improving community engagement and mobilization and improving the quality of CCM at the health systems level. Experts from organizational HQ/HICs prioritized more technical questions; the two research questions with the highest scores from this group were identifying and evaluating diagnostic tools for different cadres of CHWs and evaluating 3–day vs 5–day amoxicillin treatment in Africa. Again, this finding highlights the importance of the CHNRI exercise in allowing detections of differences in priority perceptions between groups of experts and thus allows the design of studies according to specific priorities.

While many of the high–ranking questions in the CHNRI exercise mirror those proposed by the CCM ORG [11,12], there were some notable differences. Of the top 10 research questions, questions #1, 5, 8, and 9 were generated by the CHNRI exercise and were not present in the CCM ORG’s original list of research questions. The research question that scored the second highest in the importance/potential impact category was also generated through the CHNRI exercise. For the research questions that were present in the CCM ORG’s original list, the use of the CHNRI method to systematically rank the research priorities against pre–set criteria allowed further exploration of the strengths and weaknesses of each proposed research question and lending credibility to the findings.

To our knowledge, our exercise is the first to stratify analysis based on participants’ location. The weak to moderate correlation between participants from HQ/HIC group and the LMIC group reveals that while there is some consensus on priorities, there is significant divergence that requires further examination. As it is often those working in organizational HQs or HICs who are responsible for setting the research agenda, the disconnect between their priorities and those of in–country or regional counterparts requires careful consideration. Although the differences could reflect different characteristics and interests between both groups, it could also indicate a larger problem. While we cannot say which groups’ opinion on research priorities is has more utility, the discordance is important to highlight to donors and researchers when making decisions on which priorities to fund.

In addition to displaying the research priorities in a particular area, a secondary goal of research priority setting exercises is to stimulate interest and funding for research in that area. We are currently aware of two studies are currently being designed to answer research questions from this exercise. The first is a multi–site study that will explore strategies to improve supervision, retention and motivation of CHWs (questions #2 and 14 overall) and the second will study the effects of adding IYCF counselling skills to CHWs’ workload (question #8 overall) (our unpublished data).

iCCM is capable of reducing a substantial number of unnecessary childhood deaths due to pneumonia, diarrhoea and malaria and improving equity in health care access for poor, rural and hard–to–reach communities. We hope that the results of our exercise will continue to direct and assist funders, policy–makers, program managers and researchers to identify research priorities, their potential strengths and weaknesses, and to stimulate interest and in furthering the iCCM research agenda.
