# Supplementary material for: Setting global research priorities for integrated community case management (iCCM): Results from a CHNRI (Child Health and Nutrition Research Initiative) exercise
Source: J Glob Health. 2014 Dec;4(2):020413. doi: 10.7189/jogh.04.020413 (PMC4267102; doi:10.7189/jogh.04.020413)
Supplement: Online Supplementary Document [file jogh-04-020413-s001.pdf]

Online Supplementary Document

Wazny et al. Setting global research priorities for integrated community case management (iCCM): Results from a CHNRI (Child Health and Nutrition Research Initiative) exercise.
J Glob Health 2014;4:020413

Table S1. List of Participants and Categorization

Name	Location	Categorization	Institution
Elsheik Abdalla	South Sudan	LMIC	International Rescue Committee (IRC)
Emmanuel Akach	Kenya	LMIC	African Medical and Research Foundation (AMREF)
Caroline Akim	Tanzania	LMIC	Maternal and Child Health Integrated Program (MCHIP)
Sarah Andersson	USA	HQ/HIC	John Snow, Inc. (JSI)
Wulleta Betemariam	Ethiopia	LMIC	John Snow, Inc. (JSI)
Zulfiqar Bhutta	Canada/Pakistan	HQ/HIC	The Hospital for Sick Children & Aga Khan University
Jennifer Bryce	USA	HQ/HIC	Johns Hopkins University
Tesfaye Bulto	Ethiopia	LMIC	US Agency for International Development (USAID)
Elizabeth Burleigh	USA	HQ/HIC	John Snow, Inc. (JSI)
Volkan Cakir	Madagascar	LMIC	Research Triangle Institute (RTI)
Jean Capps	USA	HQ/HIC	Independent
Christina Cardemil	USA	HQ/HIC	US Centers for Disease Control and Prevention (CDC)
Yasmin Chandani	USA	HQ/HIC	John Snow, Inc. (JSI)
Marilu Chiang	India	HQ/HIC	Clinton Health Access Initiative (CHAI)
Boniface Chiluba	Zambia	LMIC	Churches Health Association of Zambia (CHAZ)
Tedbab Degefie	Ethiopia	LMIC	United Nations Children's Fund (UNICEF)
Theresa Diaz	USA	HQ/HIC	United Nations Children's Fund (UNICEF)
Tanya Doherty	South Africa	LMIC	Medical Research Council
Shannon Downey	USA	HQ/HIC	CORE Group
M. James Eliades	USA	HQ/HIC	Columbia University
Solomon Emyu	Ethiopia	LMIC	World Health Organization (WHO)
Olivier Fontaine	France	HQ/HIC	Formerly World Health Organization (WHO)
Ciro Franco	USA	HQ/HIC	Management Sciences for Health (MSH)
Catherine Gbozee	Liberia	LMIC	John Snow, Inc. (JSI)
Joby George	Malawi	LMIC	Save the Children

Kate Gilroy	USA	HQ/HIC	Independent
Amy Ginsburg	USA	HQ/HIC	Program for Appropriate Technology in Health (PATH)
Nike Grange	Nigeria	LMIC	Otunba Tunwase Pediatric Center and Institute for Child Health
Tanya Guenther	USA	HQ/HIC	Save the Children, USA
John Gyapong	Ghana	LMIC	University of Ghana
Davidson Hamer	Zambia/USA	HQ/HIC	Boston University
Karin Kallander	Sweden	HQ/HIC	Malaria Consortium
Gagik Karapetyan	USA	HQ/HIC	World Vision
Assaye Kassie	Zimbabwe	LMIC	United Nations Children's Fund (UNICEF)
Dyness Kasungami	USA, Zambia	HQ/HIC	United Nations Children's Fund (UNICEF)
Troy Jacobs	USA	HQ/HIC	US Agency for International Development (USAID)
Yolanda Barbera Lainez	USA	HQ/HIC	International Rescue Committee (IRC)
Victor Lara	USA	HQ/HIC	Population Services International (PSI)
Zohra Lassi	Pakistan	LMIC	Aga Khan University (AKU)
Hailemariam Legesse	Ethiopia	LMIC	United Nations Children's Fund (UNICEF)
David Marsh	USA	HQ/HIC	Save the Children, USA
Ziaul Matin	Bangladesh	LMIC	United Nations Children's Fund (UNICEF)
Laura Miller	Sierra Leone	LMIC	International Rescue Committee (IRC)
Amina Issa Mohamud	South Sudan	LMIC	International Rescue Committee (IRC)
Golam Mothabbir	Bangladesh	LMIC	Save the Children
Irma Montes	Nicaragua	LMIC	Save the Children
Humphreys Nsona	Malawi	LMIC	Ministry of Health
Florence Nyangara	USA	HQ/HIC	Inner City Fund (ICF) and Maternal and Child Health Integrated Program (MCHIP)
Charles Ocan	Uganda	LMIC	Save the Children
Lolade Oseni	Malawi	LMIC	Jhpiego
Franco Pagnoni	Switzerland	HQ/HIC	World Health Organization Global Malaria Program (WHO GMP)
Luwei Pearson	Ethiopia	LMIC	United Nations Children's Fund (UNICEF)
Henry Perry	USA	HQ/HIC	Johns Hopkins University
Stefan Peterson	Sweden	HQ/HIC	Karolinska Institutet
Shamim Qazi	Pakistan	HQ/HIC	World Health Organization (WHO)
Robertine Rahelimalala	Madagascar	LMIC	John Snow, Inc. (JSI)
Dixmer Rivera	Nicaragua	LMIC	Save the Children
Alfonso Rosales	USA	HQ/HIC	World Vision
Kerry Ross	USA	HQ/HIC	Maternal and Child Health Integrated Program (MCHIP)
Elizeus Rutebemberwa	Uganda	LMIC	Makerere University

Salim Sadruddin	Canada	HQ/HIC	Save the Children, USA
Laila Salim	Canada	HQ/HIC	Save the Children, Canada
Budhi Setiawan	Indonesia	LMIC	United Nations Children’s Fund (UNICEF)
Sodoimon Sirima	Burkina Faso	LMIC	Ministry of Health
Sajid Soofi	Pakistan	LMIC	Aga Khan University (AKU)
Eric Starbuck	USA	HQ/HIC	Save the Children
Sylla Thiam	Kenya	HQ/HIC	African Medical and Research Foundation (AMREF)
Peter Waiswa	Uganda	LMIC	Makerere University
Karen Waltensperger	South Africa	LMIC	Save the Children
Emmanuel Wansi	USA	HQ/HIC	Independent
Erica Wetzler	Mozambique	LMIC	Save the Children
Cathy Wolfheim	Switzerland	HQ/HIC	Independent
Kojo Yeboah-Antwi	USA	HQ/HIC	Boston University
Jean Christian Youmba	Cameroon	LMIC	Association Camerounaise pour le Marketing Social (ACMS)
Mark Young	USA	HQ/HIC	United Nations Children’s Fund (UNICEF)

Table S2. Overall Rank and Research Priority Scores

Overall Rank	Research Question	Criterion 1: Answerability	Criterion 2: Research Feasibility	Criterion 3: Deliverability	Criterion 4: Importance/ Potential Impact	Research Priority Score (RPS)	Average Expert Agreement (AEA)
1	Assess perceptions of beneficiaries and levels of community satisfaction in CHWs capacity to diagnose and treat sick child (with malaria, pneumonia, diarrhea and severe malnutrition) at the community level.	0.88	0.96	0.95	0.78	89.31	0.84
2	Identify and evaluate strategies for retention and motivation of CHWs.	0.85	0.94	0.85	0.92	89.08	0.86
3	Identify and evaluate strategies for improving referral between communities and health facilities, including referral compliance.	0.84	0.94	0.89	0.89	88.94	0.84
4	Identify determinants of non-use of iCCM services by caretakers and develop strategies to increase the uptake of iCCM.	0.84	0.95	0.91	0.86	88.89	0.84
5	Identify and evaluate new diagnostic tools for improved classification of pneumonia (i.e. different ARI timers, respiratory counting beads, etc.) at the community level that are most appropriate for various cadres.	0.87	0.90	0.85	0.93	88.83	0.85
6	Evaluate the effectiveness of 3-day versus 5-day amoxicillin treatment regimens in Africa	0.83	0.88	0.92	0.91	88.61	0.84
7	Identify and evaluate innovative strategies to improve community engagement and mobilization for CCM.	0.78	0.96	0.89	0.87	87.49	0.83
8	Evaluate the feasibility, effectiveness and impact of adding community-based infant and young child feeding (cIYCF) counseling skills to the CHW workload.	0.87	0.91	0.85	0.86	87.26	0.82
9	Identify the primary barriers to CHW supervision and develop and evaluate strategies to motivate CHW supervisors to provide continuous support to CHWs.	0.81	0.93	0.86	0.90	87.18	0.82
10	What is the impact of pre-referral antibiotics on treatment outcomes of possible serious bacterial infections?	0.83	0.88	0.87	0.89	86.52	0.80
11	Assess perceptions, understanding and motivating factors for caregivers on the need for prompt treatment for the sick child.	0.85	0.97	0.86	0.78	86.41	0.82
12	What is the impact of iCCM on health facility worker workload, by disease?	0.82	0.95	0.89	0.80	86.37	0.81
13	Develop and evaluate strategies (for example, innovative packaging of	0.85	0.89	0.86	0.83	86.00	0.81

	drugs) to improve compliance and uptake of treatment.						
14	Identify and evaluate strategies to improve supervision and quality of care using mHealth technology.	0.86	0.91	0.80	0.87	85.85	0.81
15	Identify and evaluate effective and feasible strategies for maintaining quality of case management by CHWs.	0.77	0.90	0.83	0.93	85.56	0.82
16	Identify and evaluate strategies for, and costs of, supervising the CHW supervisor.	0.81	0.93	0.88	0.80	85.35	0.79
17	Develop and evaluate strategies for using mHealth technology to improve drug supply and logistics for the CHWs.	0.87	0.87	0.79	0.88	85.28	0.79
18	Evaluate the impact of iCCM on equity in access and use of basic health services.	0.79	0.90	0.88	0.83	85.14	0.80
19	Identify and evaluate the effectiveness and cost of various incentive schemes and strategies for CHWs.	0.81	0.88	0.81	0.89	84.81	0.79
20	Identify and evaluate strategies to improve integration of iCCM logistics (diagnostics and drug supply) to the central procurement and supply system at the community level.	0.80	0.87	0.83	0.87	84.41	0.79
21	Evaluate the effectiveness and feasibility of delivering treatment for Severe Acute Malnutrition (SAM) through iCCM.	0.82	0.84	0.81	0.90	84.08	0.80
22	Identify and evaluate determinants of quality of CCM services, including characteristics of health systems (and supporting environment) that are most important for delivering high quality iCCM programs at-scale with limited external support.	0.74	0.86	0.84	0.89	83.22	0.77
23	Evaluate the impact of involving community structures (e.g. community health committees, traditional leaders, opinion and influential leaders) in enhancing the sustainability and accountability of iCCM.	0.80	0.89	0.84	0.80	83.05	0.78
24	What are the feasibility, impact and costs of adding newborn care (including PNS, home visits, treatment of infection and Caring for the Newborn and Children in the Community) to the iCCM package?	0.78	0.86	0.77	0.90	82.74	0.76
25	Evaluate models for iCCM that are most appropriate for urban settings.	0.83	0.86	0.82	0.80	82.74	0.76
26	Identify and evaluate effective delivery strategies for iCCM in marginalized populations or areas.	0.74	0.87	0.84	0.86	82.66	0.74
27	Will the rapid scale-up of RDTs for malaria at the community level have the unintended consequence of overuse of antibiotics for malaria?	0.81	0.87	0.83	0.79	82.59	0.76
28	Evaluate the impact of CHW gender on CCM delivery and utilization.	0.83	0.93	0.85	0.68	82.31	0.76
29	Determine the optimal case-load and catchment area for CHWs in high, medium and low population density that balances maintaining quality of	0.72	0.86	0.84	0.85	81.78	0.76

	care and prevention of loss of skills.						
30	Develop strategies to ensure acceptable quality of care in hard-to-reach settings where less literate CHWs are the most appropriate providers by use of a specifically designed package of training, supervision and tools.	0.71	0.85	0.83	0.88	81.43	0.76
31	Does the use of mHealth technology lead to an improvement of iCCM service delivery, for example by providing back-up for CHWs when they encounter difficult cases or for defaulter tracking?	0.84	0.84	0.77	0.80	81.42	0.73
32	Develop and evaluate strategies to improve caregiver acceptance of not receiving antimalarials or antibiotics for negative RDT result and cough without fast breathing, respectively.	0.77	0.89	0.86	0.73	81.32	0.74
33	Identify and evaluate the elements of a comprehensive community support structure best suited to mobilize and sustain demand for CCM services in various contexts.	0.74	0.86	0.78	0.87	81.10	0.73
34	What behavior change strategies are most effective in creating demand and managing expectations of parents?	0.72	0.86	0.82	0.84	81.09	0.75
35	Evaluate the impact of CHW activity on the incidence of severe cases of malaria, pneumonia, diarrhea and malnutrition in sites where CCM is implemented.	0.79	0.79	0.85	0.82	80.96	0.75
36	Can CHWs effectively treat WHO-defined severe pneumonia?	0.80	0.84	0.74	0.85	80.76	0.77
37	What basic level of capacity and resources are needed at first level health facilities in areas implementing iCCM?	0.77	0.91	0.84	0.69	80.39	0.74
38	Does giving a non-therapeutic intervention (i.e. honey and lemon or paracetamol) to children with mild cases who do not need treatment result in higher parent satisfaction?	0.83	0.87	0.86	0.66	80.32	0.74
39	Evaluate the effectiveness of using mHealth applications for CHWs in improving RDT results and rational use of drugs.	0.83	0.86	0.74	0.79	80.28	0.73
40	Evaluate the effectiveness and feasibility of CHWs' use of pulse oximetry to identify children with severe pneumonia.	0.85	0.79	0.69	0.87	79.84	0.75
41	Evaluate the effectiveness of adding multimedia to training materials during field supervision and health facility mentoring sessions to improve quality of clinical mentoring and non-clinical supervision.	0.88	0.83	0.73	0.75	79.82	0.72
42	How does the selection proves of Community Based Distributors or CHWs facilitate or hinder iCCM implementation?	0.77	0.89	0.86	0.66	79.50	0.73
43	Identify the minimum capacity building package necessary to impart the appropriate knowledge and skills amongst CHWs (both literate and less literate) to deliver optimal quality care at the community or primary	0.71	0.84	0.84	0.77	79.17	0.71

	health care level.						
44	Assess the impact of various forms of financial incentives (e.g. cash transfers, microcredit, vouchers) for extremely poor households on careseeking and improved access to quality CCM services.	0.80	0.86	0.73	0.77	79.09	0.72
45	Compare the cost of implementing iCCM versus the cost of services provided only through health facilities in different contexts (e.g. rural, urban/slums, remote areas, pastoralists).	0.75	0.81	0.80	0.80	78.95	0.72
46	Can CHWs effectively manage pneumonia in newborns (0-2 months)?	0.75	0.79	0.73	0.89	78.80	0.72
47	Evaluate cost and cost-benefits of different scalable iCCM delivery models.	0.73	0.77	0.81	0.83	78.23	0.70
48	What are the critical conditions that need to be met to attain and maintain high effective coverage (i.e. a high proportion of the actual number of cases in a population receiving appropriate care) for treatment of childhood illness in iCCM programs operating at-scale?	0.71	0.80	0.78	0.82	77.90	0.71
49	Identify and evaluate scalable strategies to substantially and sustainably improve the quality of iCCM services delivered by private providers and drug retailers in both rural and urban settings.	0.71	0.80	0.77	0.83	77.58	0.70
50	Can CHWs effectively manage sepsis in newborns (0-2 months)?	0.75	0.76	0.73	0.84	76.91	0.70
51	Evaluate the cost and cost-effectiveness of iCCM programs with carrying scope of illness, geographic settings, population density, disease epidemiology, etc.	0.65	0.78	0.76	0.86	76.55	0.72
52	Develop safe and effective treatment strategies in settings where referral is not possible.	0.62	0.79	0.73	0.90	75.88	0.68
53	Identify and evaluate the effectiveness and cost of mHealth applications to help family members recognize disease, seek care and adhere to treatment recommendations.	0.78	0.78	0.68	0.78	75.64	0.67
54	Evaluate the effect of iCCM on antimicrobial resistance.	0.77	0.72	0.73	0.80	75.39	0.68
55	What is the effect on motivation and quality of services offered by CHWs of integrating CHW cadre into national human resource plans, including mechanisms for raises/promotions, career advancement, etc.?	0.67	0.79	0.69	0.83	74.41	0.67
56	What are the feasibility, effect, impact and costs of adapting the CCM package for more timely identification of children under five who are HIV-exposed and HIV-positive in high-prevalence settings for HIV?	0.69	0.76	0.71	0.78	73.38	0.63
57	Identify and evaluate an algorithm to distinguish infants and children with severe disease who can be treated at home versus those who must be referred to the hospital.	0.65	0.76	0.74	0.77	73.30	0.65

58	What effect does increasing complexity of illness have on the quality of care delivered through iCCM (particularly in young children or children with malnutrition and/or HIV)?	0.65	0.73	0.70	0.77	71.39	0.62
59	Evaluate whether CHWs can safely and appropriately manage severe pneumonia without hypoxia in Africa.	0.68	0.69	0.66	0.81	70.97	0.63
60	What proportion of CHWs' time should be spent on health promotion versus curative services?	0.68	0.79	0.73	0.63	70.88	0.64
61	Are there criteria for 'moderately ill' children by syndrome, and could children satisfying these criteria be managed in the community (similarly to 'chest indrawing pneumonia' that has been proven to be manageable at the community level)?	0.53	0.67	0.65	0.73	64.58	0.54

Table S3. Research Priority Scores for each Research Priority by Participant Category

Overall Rank	Research Question	Overall Research Priority Score (RPS)	HQ-level Research Priority Score	HQ Rank	Country-level RPS	Country-level rank
1	Assess perceptions of beneficiaries and levels of community satisfaction in CHWs capacity to diagnose and treat sick child (with malaria, pneumonia, diarrhea and severe malnutrition) at the community level.	89.31	86.90	3	91.79	6
2	Identify and evaluate strategies for retention and motivation of CHWs.	89.08	85.89	10	92.27	4
3	Identify and evaluate strategies for improving referral between communities and health facilities, including referral compliance.	88.94	86.86	4	91.12	9
4	Identify determinants of non-use of iCCM services by caretakers and develop strategies to increase the uptake of iCCM.	88.89	85.13	14	92.61	2
5	Identify and evaluate new diagnostic tools for improved classification of pneumonia (i.e. different ARI timers, respiratory counting beads, etc.) at the community level that are most appropriate for various cadres.	88.83	91.09	1	86.34	21
6	Evaluate the effectiveness of 3-day versus 5-day amoxicillin treatment regimens in Africa	88.61	89.83	2	87.23	17
7	Identify and evaluate innovative strategies to improve community engagement and mobilization for CCM.	87.49	82.85	21	92.19	5
8	Evaluate the feasibility, effectiveness and impact of adding community-based infant and young child feeding (cIYCF) counseling skills to the CHW workload.	87.26	83.43	19	91.29	8
9*	Identify the primary barriers to CHW supervision and develop and evaluate strategies to motivate CHW supervisors to provide continuous support to CHWs.	87.18	81.51	26	93.10	1
10	What is the impact of pre-referral antibiotics on treatment outcomes of possible serious bacterial infections?	86.52	86.03	8	87.06	19
11	Assess perceptions, understanding and motivating factors for caregivers on the need for prompt treatment for the sick child.	86.41	83.86	17	89.03	11
12	What is the impact of iCCM on health facility worker workload, by disease?	86.37	81.15	30	91.46	7
13	Develop and evaluate strategies (for example, innovative packaging of drugs) to improve compliance and uptake of treatment.	86.00	85.28	13	86.80	20

14	Identify and evaluate strategies to improve supervision and quality of care using mHealth technology.	85.85	85.43	12	86.26	22
15	Identify and evaluate effective and feasible strategies for maintaining quality of case management by CHWs.	85.56	82.34	24	88.74	12
16	Identify and evaluate strategies for, and costs of, supervising the CHW supervisor.	85.35	82.49	23	88.36	13
17	Develop and evaluate strategies for using mHealth technology to improve drug supply and logistics for the CHWs.	85.28	83.45	18	87.25	16
18	Evaluate the impact of iCCM on equity in access and use of basic health services.	85.14	85.94	9	84.39	27
19	Identify and evaluate the effectiveness and cost of various incentive schemes and strategies for CHWs.	84.81	80.47	32	89.51	10
20	Identify and evaluate strategies to improve integration of iCCM logistics (diagnostics and drug supply) to the central procurement and supply system at the community level.	84.41	86.25	7	82.60	36
21	Evaluate the effectiveness and feasibility of delivering treatment for Severe Acute Malnutrition (SAM) through iCCM.	84.08	81.30	28	87.07	18
22*	Identify and evaluate determinants of quality of CCM services, including characteristics of health systems (and supporting environment) that are most important for delivering high quality iCCM programs at-scale with limited external support.	83.22	73.52	54	92.55	3
23	Evaluate the impact of involving community structures (e.g. community health committees, traditional leaders, opinion and influential leaders) in enhancing the sustainability and accountability of iCCM.	83.05	81.17	29	85.08	26
24	What are the feasibility, impact and costs of adding newborn care (including PNS, home visits, treatment of infection and Caring for the Newborn and Children in the Community) to the iCCM package?	82.74	80.31	33	85.29	25
25	Evaluate models for iCCM that are most appropriate for urban settings.	82.74	79.44	36	86.15	23
26*	Identify and evaluate effective delivery strategies for iCCM in marginalized populations or areas.	82.66	76.97	44	88.11	14
27**	Will the rapid scale-up of RDTs for malaria at the community level have the unintended consequence of overuse of antibiotics for malaria?	82.59	85.63	11	78.78	44
28	Evaluate the impact of CHW gender on CCM delivery and utilization.	82.31	81.95	25	82.67	35

29	Determine the optimal case-load and catchment area for CHWs in high, medium and low population density that balances maintaining quality of care and prevention of loss of skills.	81.78	80.65	31	82.96	32
30	Develop strategies to ensure acceptable quality of care in hard-to-reach settings where less literate CHWs are the most appropriate providers by use of a specifically designed package of training, supervision and tools.	81.43	77.33	43	85.65	24
31	Does the use of mHealth technology lead to an improvement of iCCM service delivery, for example by providing back-up for CHWs when they encounter difficult cases or for defaulter tracking?	81.42	79.17	37	83.92	29
32**	Develop and evaluate strategies to improve caregiver acceptance of not receiving antimalarials or antibiotics for negative RDT result and cough without fast breathing, respectively.	81.32	84.89	15	77.72	48
33*	Identify and evaluate the elements of a comprehensive community support structure best suited to mobilize and sustain demand for CCM services in various contexts.	81.10	74.50	50	88.03	15
34	What behavior change strategies are most effective in creating demand and managing expectations of parents?	81.09	79.45	35	82.76	34
35	Evaluate the impact of CHW activity on the incidence of severe cases of malaria, pneumonia, diarrhea and malnutrition in sites where CCM is implemented.	80.96	78.96	38	83.10	7
36**	Can CHWs effectively treat WHO-defined severe pneumonia?	80.76	86.51	6	74.50	56
37	What basic level of capacity and resources are needed at first level health facilities in areas implementing iCCM?	80.39	78.56	40	82.26	38
38**	Does giving a non-therapeutic intervention (i.e. honey and lemon or paracetamol) to children with mild cases who do not need treatment result in higher parent satisfaction?	80.32	84.01	16	76.32	53
39	Evaluate the effectiveness of using mHealth applications for CHWs in improving RDT results and rational use of drugs.	80.28	83.21	20	77.15	50
40**	Evaluate the effectiveness and feasibility of CHWs' use of pulse oximetry to identify children with severe pneumonia.	79.84	86.57	5	72.35	57
41	Evaluate the effectiveness of adding multimedia to training materials during field supervision and health facility mentoring sessions to improve quality of clinical mentoring and non-clinical supervision.	79.82	82.75	22	76.67	52
42	How does the selection proves of Community Based Distributors or CHWs facilitate or hinder iCCM implementation?	79.50	74.89	48	84.19	28

43	Identify the minimum capacity building package necessary to impart the appropriate knowledge and skills amongst CHWs (both literate and less literate) to deliver optimal quality care at the community or primary health care level.	79.17	76.02	45	82.42	37
44	Assess the impact of various forms of financial incentives (e.g. cash transfers, microcredit, vouchers) for extremely poor households on careseeking and improved access to quality CCM services.	79.09	79.53	34	78.64	45
45	Compare the cost of implementing iCCM versus the cost of services provided only through health facilities in different contexts (e.g. rural, urban/slums, remote areas, pastoralists).	78.95	78.57	39	79.47	41
46	Can CHWs effectively manage pneumonia in newborns (0-2 months)?	78.80	81.44	27	76.01	54
47	Evaluate cost and cost-benefits of different scalable iCCM delivery models.	78.23	73.73	53	82.78	33
48*	What are the critical conditions that need to be met to attain and maintain high effective coverage (i.e. a high proportion of the actual number of cases in a population receiving appropriate care) for treatment of childhood illness in iCCM programs operating at-scale?	77.90	72.05	56	83.76	30
49	Identify and evaluate scalable strategies to substantially and sustainably improve the quality of iCCM services delivered by private providers and drug retailers in both rural and urban settings.	77.58	75.08	47	80.22	39
50	Can CHWs effectively manage sepsis in newborns (0-2 months)?	76.91	77.92	41	75.87	55
51	Evaluate the cost and cost-effectiveness of iCCM programs with carrying scope of illness, geographic settings, population density, disease epidemiology, etc.	76.55	74.16	51	79.15	42
52	Develop safe and effective treatment strategies in settings where referral is not possible.	75.88	74.69	49	77.10	51
53	Identify and evaluate the effectiveness and cost of mHealth applications to help family members recognize disease, seek care and adhere to treatment recommendations.	75.64	73.93	52	77.28	49
54	Evaluate the effect of iCCM on antimicrobial resistance.	75.39	70.97	57	79.96	40
55	What is the effect on motivation and quality of services offered by CHWs of integrating CHW cadre into national human resource plans, including mechanisms for raises/promotions, career advancement, etc.?	74.41	70.48	58	78.18	47
56	What are the feasibility, effect, impact and costs of adapting the CCM package for more timely identification of children under five who are	73.38	68.35	59	78.32	46

	HIV-exposed and HIV-positive in high-prevalence settings for HIV?					
57	Identify and evaluate an algorithm to distinguish infants and children with severe disease who can be treated at home versus those who must be referred to the hospital.	73.30	75.31	46	71.08	58
58	What effect does increasing complexity of illness have on the quality of care delivered through iCCM (particularly in young children or children with malnutrition and/or HIV)?	71.39	73.47	55	69.39	59
59	Evaluate whether CHWs can safely and appropriately manage severe pneumonia without hypoxia in Africa.	70.97	77.44	42	63.42	61
60	What proportion of CHWs' time should be spent on health promotion versus curative services?	70.88	63.22	61	79.08	43
61	Are there criteria for 'moderately ill' children by syndrome, and could children satisfying these criteria be managed in the community (similarly to 'chest indrawing pneumonia' that has been proven to be manageable at the community level)?	64.58	64.24	60	64.83	60

* Indicates one of five research questions with greatest difference in rank, ranked highly by LMIC representatives

** Indicates one of five research questions with greatest difference in rank, ranked highly by HQ/HIC representatives
